# Biopsychosocial Aspect of Patients With X-Linked Dystonia-Parkinsonism: Its Implications on Quality of Life

**DOI:** 10.7759/cureus.21699

**Published:** 2022-01-28

**Authors:** Aljeirou Alcachupas, Krisverlyn Bellosillo, Wynlee Rhm Catolico, Mark Cullen Davis, Alyssa Diaz, Yvette Karla Doyongan, Reczy Eduarte, Emerald Gersava, Mary Bernadette Intrepido, Maugri Grace Kristi Laluma, Candra Carmelli Lavalle, Jeffrey Jr Millan

**Affiliations:** 1 College of Medicine, West Visayas State University, Iloilo City, PHL

**Keywords:** dystonic disorder, lived experiences, self concept, quality of life, x-linked dystonia parkinsonism

## Abstract

Objective: This study aims to describe the demographic profile in terms of age, marital status, annual family income, and educational attainment; to describe the physical, psychological, and social manifestations; to determine and describe coping mechanisms; to determine the goals, aspirations, and needs; and to determine the interaction and impact of the lived experiences on the quality of life of X-linked dystonia-parkinsonism (XDP) patients.

Methods: This qualitative-phenomenological study was conducted in the island of Panay. Purposive sampling was utilized. The researchers utilized in-depth interviews, observation, and triangulation as part of the data collection methods. The data were transcribed verbatim, kept for content analysis, and coded in their appropriate cell categories after themes were identified.

Results: Ten male patients who were residents of Panay and aged 30-65 years old participated in this study. Disease manifestations included limb dystonia, blepharospasm, truncal torsion, oromandibular symptoms, torticollis, and dysphonia, contributing to limitations in performing activities of daily living. Denial was the most common initial reaction after being diagnosed with XDP. Social manifestations were greatly affected by family and community. Money and medications were the primary needs identified by the patients with hopes of a better future for their families. There was an overall deterioration in the quality of life of the patients.

Conclusions: XDP greatly affected the physical, psychological, and social aspects of the patients. Coping with the disease and its effects has been thought to play an important role in the perception of one’s quality of life.

## Introduction

X-linked dystonia-parkinsonism (XDP), locally known as “lubag,” is a monogenic disease with a high prevalence rate among adult men of Filipino descent, especially those with ancestries from the island of Panay. It is a movement disorder characterized by sustained or spasmodic, involuntary, patterned, and repetitive muscle contractions, which result in physical deformities that are disabling and permanent [1].

The World Health Organization defines the quality of life (QoL) as an individual’s perception of his position in life in the context of culture and value systems in which he lives in and in relation to his goals, expectations, standards, and concerns. In XDP, the physical manifestations influence the patient’s overall health, including the emotional, psychological, social, and spiritual aspects [2].

Research studies have been conducted correlating debilitating health conditions and QoL but there remains a few specific for dystonia. Of critical consideration in XDP is that the patients are afflicted in the prime of their years, the crucial time when they are on the road of building their dreams, when they have attained stability in their jobs, and when they are considered family breadwinners. The accompanying social stigma attributed to the varying mild to severely grotesque physical deformities leads to interpersonal impairments. This disease has no known cure; definite symptomatic relief could only be achieved through costly measures. The disease will predictably continue to surface among male, and rarely female, members of these families for generations [3-5].

This study aimed to describe the biopsychosocial aspect of patients with XDP and its implications on QoL. Specifically, this study sought to describe the demographic profile of patients in terms of age, marital status, annual family income, and educational attainment; to describe their physical, psychological, and social manifestations and coping mechanisms; to determine their goals, aspirations, and needs; and to explore the lived experiences of XDP patients and its impact to their QoL.

## Materials and methods

This is a phenomenological study reported through the presentation of cases. Phenomenology is a qualitative type of research employed to understand and explore the lived experiences of individuals who share the same phenomenon from a first-person perspective [6-8]. This qualitative study was conducted in the island of Panay in the Philippines. A purposive and maximal variation sampling strategy was applied. The names of the patients diagnosed with XDP were obtained from neurologists in Iloilo and Capiz. The study was approved by the West Visayas State University Unified Bio-Medical Research Ethics Review Committee (WVSU.UBRERC-2014.COM_009), and the respondents were asked to sign an informed consent form written in the local language, Hiligaynon, prior to the data gathering.

The researchers utilized in-depth interviews, observation, and triangulation to collect data. A researcher-made and expert-validated questionnaire, containing descriptive and qualitative questions, was used as a guide to attaining the study objectives. Particular importance was also given to the patients’ physical surroundings, patterns of interaction with family members, and non-verbal cues. Triangulation was done to strengthen the validity of data in terms of scope, depth, and consistency. To maintain objectivity and comprehensiveness, the following measures were undertaken: peer debriefing, familiarity, and engagement with study population, reflection and processing, and member checking [9-12].

**Figure 1:**
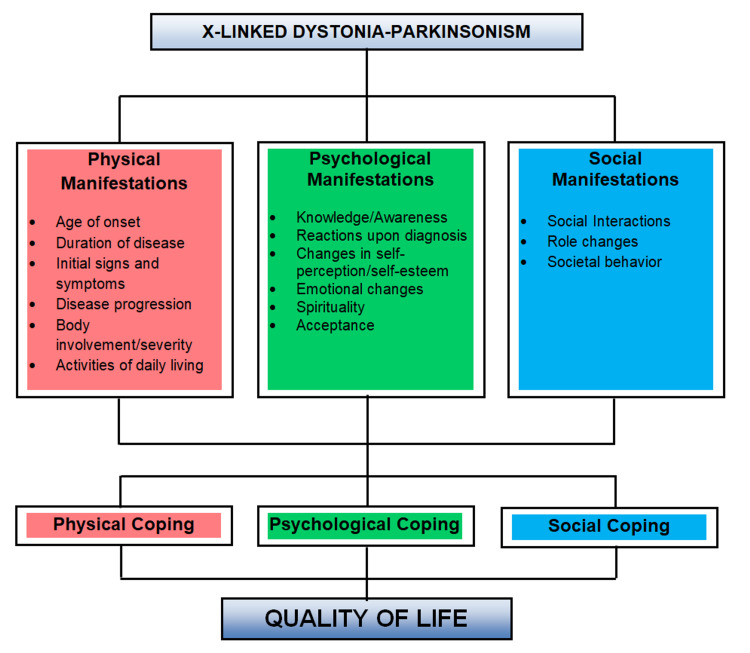
Conceptual framework This study is anchored on five theories including Engel’s Biopsychosocial, Maslow’s Hierarchy of Needs, Kubler-Ross’ Five Stages of Grief, Lazarus and Folkman’s Transaction Model, and Roy’s Adaptation Model. Common aspects were identified from these theories and grouped into categories, namely, physical, psychological, and social manifestations and aspects of coping. Recurrent themes and peculiarities were extracted from the patient interviews and were bracketed into specific categories and themes to describe the overall lived experience and quality of life of patients.

In the analysis of data, audiotapes and videotapes were listened and viewed several times to gain familiarity with what was said by the respondents. Transcription of the verbatim then commenced. The researchers looked into meaningful statements in the interviews, which reflected the participants’ experiences surrounding the phenomenon. Peculiarities and similarities were also looked into. Next, the statements were grouped by the researchers into meaningful units through meticulous coding and selective coding and were bracketed into categories and themes. Lastly, descriptions of how the phenomenon was experienced were established from these themes and an overall description of the essence of the experience was constructed (Figure 1).

## Results

Patient demographics

A total of 10 men were interviewed for this qualitative research. All were residents of Panay. Table 1 summarizes the demographic profiles of patients interviewed.

**Table 1:** Demographic profile of the respondents

Personal profile of the respondents	Frequency	Percentage (%)
Age		
30-39 years old	1	10
40-49 years old	3	30
50-60 years old	5	50
>60 years old	1	10
Age of onset of disease		
<30 years old	2	20
30-39 years old	1	10
40-50 years old	7	70
Years of manifesting the disease		
<1 year	2	20
1-5 years	5	50
6-10 years	1	10
>10 years	2	20
Educational attainment		
Elementary graduate	0	-
High school level	1	10
High school graduate	2	20
College graduate	6	60
Vocational course	1	10
Annual family income		
P 10,000-30,000	2	20
P 30,001-60,000	1	10
P 60,001-100,000	1	10
P >100,000	6	60
Address		
Capiz province	6	60
Provinces outside Capiz within Panay	4	40
Marital status		
Married	10	100
Occupation (before the disease)		
Blue collar	4	50
White collar	5	40
Pink collar	1	10

Age is an important factor to consider in describing a patient’s QoL. The age of XDP patients ranges from the early 30s to early 60s. To give more significance to the age, one would compare it with the patient’s disease onset to know how long they have endured the disease. It was noted that two of the patients’ disease onsets were in their late 20s and seven began manifesting the symptoms in their 40s. One patient, on the other hand, apparently manifested the disease much later at the age of 51. The duration of illness from the onset of the disease to the present was six months as the shortest and 14 years as the longest. More than half of the patients had the disease for one to four years, while three patients endured the disease for more than 10 years now.

These patients present with a wide array of professional and working backgrounds, ostensibly showing that the disease does not single out the poor or the rich and the educated or the illiterate. Most of the patients had worked as professionals before their disease began to establish. Most of them were forced to early retirement or quit their jobs as the symptoms gradually brought the decline of their functional abilities. Currently, one patient is still able to function as an income-generating member of his family by working as a motorized rickshaw (tricycle) driver; it was noted, however, that this patient previously worked as a seafarer, and thus experienced a step-down from his previously reported five-figure monthly salary.

Five of the patients were white-collared workers: they being a seafarer, a government employee, a teacher, an air force officer, and a general manager. Four patients were blue-collared workers: a tricycle driver who moonlights as a farmer and works in animal husbandry, one is a mechanic and carpenter, another worked as an electrician overseas and became a club bouncer upon returning to the Philippines, and one patient worked as a houseboy. One patient’s occupation falls under pink collared job: having owned and managed a fruit shake stand.

Physical examination: a general survey

Table 2 presents the summarized clinical demographic profile of the respondents. The patients possessed different body builds and body masses. What was common among the patients, especially those who had suffered dystonia for several years and the affectation is either the limb or the trunk, was that they showed an evident decreased in the subcutaneous tissue, and the muscles of tension have hypertrophied and become distinct. The veins supplying the particular limb regions also appeared distended; apparently, varicosities had also developed over time. Those with oromandibular symptoms carried a towel for their drooling. They would often cover their mouth out of habit.

**Table 2:** Clinical demographics of respondents

	Frequency	Percentage (%)
Earliest manifestation of the disease		
Limb dystonia	6	60
Blepharospasm	3	30
Truncal/lumbar involvement	1	10
Disease involvement (can overlap)		
Limb dystonia	5	50
Blepharospasm	3	30
Truncal torsion or involvement	2	20
Oromandibular symptoms	4	40
Torticollis	3	30
Dysphonia	4	40
Activities of daily living performed alone		
Ambulation	9	90
Bathing	8	80
Feeding	9	90
Dressing up	8	80
Chores and errands	3	30
Activities needing assistance (can overlap)		
Ambulation	1	10
Bathing	2	20
Feeding	1	10
Dressing up	2	20
Chores and errands	7	70
None	5	50
Primary persons assisting in activities of daily living		
Wife	5	50
Children	1	10
Parents	1	10
Self	1	10
None	2	20

Weight loss as reported was confirmed through observable loose skin and muscle wasting on other areas not affected. Only one patient was able to say that he maintained his weight and muscle mass.

Patient backstories (actual names are not used)

Big Boy previously worked at the Philippine Air Force. He retired early, long before the disease set in. Currently, he watches over his business as a shipment ticketing outlet. Among the patients, Big Boy appeared to have experienced the least change in his lifestyle instigated by the disease, though he verbalized his fears about the progression of the disease leading him to a lifetime of bed confinement.

Didoy was a workaholic. Most of his frustrations regarding his diagnosis and physical limitations branched from his inability to work and give his family their needs. Nowadays, his wife works as a rice harvester and their financial stability had been severely compromised. He verbalized that he was losing the will to live in his state. Lately, he was enlightened to speak about how he derived his hope and strength from his family.

Paul was just 29 years old when he was diagnosed with XDP and it had been a year since his diagnosis. Up until the first interview, Paul was seen to be in a state of denial. He said that his condition, which first manifested as uncontrolled closure of the left eyelid, was merely muscle spasms. His continued denial led him to vices such as alcohol and drugs. He was lately petitioned for deep brain stimulation surgery in Germany for the treatment of XDP. Paul hoped for the eventual cure of his symptoms.

Samuel had the earliest disease onset. He is now 40 years old and had been battling dystonia for almost 12 years. He currently lives with his mother and two of his children. According to his mother, he attempted suicide twice, which he denied during the interview. Even if Samuel claimed that he had come into terms with the chronicity and the incurability of the disease, he hoped that his pains and sufferings will end soon in the form of death.

One of the most peculiar presentations of dystonia was seen in the case of Lars, whose change of voice was brought about by the onset of torticollis. His voice sounded high and almost female. Lars is 42 years old and had been suffering from XDP for two years. Currently, his wife works abroad to provide for their family while he stays at home with their children. He often gets emotional when discussing his illness and stated that he felt like a burden to his family, especially his wife.

Junior worked as a tricycle driver and remained the only patient in this research who still earned an income. The biggest effect the disease had on Junior was on his ability to communicate. Upon the second visit, his speech seemed harder to comprehend. Junior experienced difficulties with relationships especially in his marriage because of the disease. However, he continued to work for a living so that his children can go to school and graduate.

Danny, a mechanic and a craftsman, suffered XDP for almost four years now. His mechanic shop was situated outside of his house where he used to work together with his sons. Diagnosed at the age of 51, the 55-year-old father was no stranger to dystonia as he had relatives afflicted by the disease. Currently, he still performs menial tasks and guides his son. However, he could no longer do carpentry because of his paroxysmal hand spasms.

The oldest person in the study living with dystonia is Colas. He was 62 years old and previously worked in a bank as a general manager. Colas was considered well-off; he owned an electronic wheelchair that he proudly showed around. Regarding his perspective of his current QoL, he knows that he had lived a good life even with the disease in picture and believes that he had done everything that he can.

The shortest duration of disease manifestation was six months. Ollie had blepharospasm and this prompted him to see an ophthalmologist. He worked as a government employee of the Department of Environment and Natural Resources (DENR). As for his QoL, he purported that there had certainly been no changes in his functioning capacity but frequently worries about what the future will bring.

## Discussion

XDP is an adult-onset, sex-linked disorder; the male-to-female ratio is 99:1 [4]. Ten patients with XDP were interviewed and observed for this qualitative research. All were male residents of the island of Panay. Their ages ranged from the early 30s to early 60s, with the disease age of onset ranging from late 20s to 40s. On the basis of the duration of the disease from the onset, the patients were noted to have the disease for six months to 14 years. Patients had colorful working experiences and different educational backgrounds ostensibly showing that the disease does not single out the poor or the rich and the educated or the illiterate. Table 3 enumerates the psychological and social themes and patterns in relation to perceived QoL, and Table 4 summarizes the coping mechanism themes and patterns derived from the interview.

**Table 3:** Psychological and social themes and patterns QoL, quality of life.

Physical manifestations	Psychological manifestations	Social impact of the disease	Quality of life
Disease timeline			
The onset of manifestations: symptoms mimicking muscle spasms and mild stroke	Silent onset, gradual progression	Able to function role as a husband and father	Physically independent
	Disease unawareness	Breadwinner	Financially secured
			Contentment
			Had concrete plans for the future
			Nonmedical interventions sought
			Good QoL
Disease confirmation and diagnosis	Shock	Isolation	Emotional instability and family strain
	Denial and disbelief	Needy for attention and reassurance	Financial assistance needed
			Future appeared uncertain
			Professional medical opinion challenged
Manifestation of the disease through the years			
<1 year: minimal disease involvement, facial affectations	Unknown fears of the future	More family-oriented	Physically able and independent
			Early retirement
			Worried about financial future
			Adherent to regular check-ups
1-10 years: progressive segmental dystonia involving more than one area of the body	Anger and hostility	Homebound	Draining financial resources
	Bargaining and increased spirituality	Public isolation and limited interactions	Relationships challenged
	Depression and suicidal ideations	Variable effects on family relationships	Limited activities
	Irritability		Maximized remaining physical capacities
	Frustration		Seeking full self-acceptance of condition
			Declining perception of QoL
			Compliant to medical treatment
>10 years: generalized dystonia, confinement to bed or wheelchair	Acceptance	Family contentment and peace	Well-adjusted to life
	Exhaustion	Roles established	Improved and enlightened perception of the QoL
	Yearning for death		Death is welcomed
			Fixed perception of the future
Body involvement of disease			
Focal to segmental: limb dystonia, blepharospasm, truncal torsion, oromandibular, torticollis, and dysphonia	Deteriorating self-esteem	Relationships unstable	Progression of disease increased physical limitations
	Fear for impeding uselessness	Family member assistance	Poor perception of QoL
		Begun to isolate self	Adherence and dependence on medications
			Draining financial resources
Generalized: affects mostly all regions of the body	Depression intensified	Isolation	Fully dependent
	Gradual acceptance	Home-based recreations	Well-adjusted to physical limitations
		Dependent role established	Contentment and acceptance as the only option
			Appreciative of the present
Disease's effects on activities of daily living			
Independent: able to ambulate, bathe, feed, dress up, do chores and errands	Independence esteemed	No significant changes in relationships	Fear for imminent disability caused the shaky perception of QoL
	Fear for total dependence		Independence valued
Needs assistance: cannot perform activities of daily living Independently	Frustrations	Assumed the role of family burden	Poor QoL
	Feeling of worthlessness		Complete dependence on the primary caregiver
			Reliant on medications for improvement of symptoms
Knowledge of the disease			
With prior knowledge: with knowledge of the existence of the disease or had witnessed the course of the disease from family members	Denial	Projected anger	Anticipated decline in QoL
	Anger	Social isolation	Anticipated future loss of resources
	Straightforward acceptance	Sustained relations	Family dynamic changes
No prior knowledge or awareness	Disbelief	Unchanged relations	Abrupt change in future plans
	Feared disease progression		Declining QoL

**Table 4:** Coping mechanism themes and patterns

Physical coping
Consultations sought
Medication intake
Botox injections
Faith healers visit
Regular check-ups
Psychological coping
The need to share the burden and the need for reciprocation
Expression through words
Emotional reciprocity expectations
Emotional connections re-established
The healing in tears and prayers
Crying
Prayer
Finding effective diversion tactics
Entertaining recreations
Vices
Hobby remodeling
Suicide contemplations
Acceptance and coping
Time as vehicle
Individualized experience
Social coping
Role acclimatization: giving in to disability
Self-reliance
Family as strength
The wife: a maker or a breaker
Spouse as pillar
A picture of a father
Role identification
Keeping the social circle
Community support
People stigma
Societal expectations

Physical, psychological, and social manifestations

Physical Manifestations

XDP is a chronic progressive disease with a variable clinical onset. Six patients reported manifesting limb dystonia as the first symptom, which appeared as twitching, shaking, stiffening, numbness, or spasmodic movement of one hand, arm, or leg, or in some instances, both extremities. These findings were consistent with the previous studies wherein 19 out of 28 patients initially presented with limb dystonia [4].

Trudging on a tiptoe: This theme explains early manifestations and disease progression. The symptom onset was not described as entirely alarming as such events would raise major red flags. The patients, most often, dismissed the symptoms as a temporary side effect of previous hard labor or curable disease, rarely entertaining the chronicity and gravity of the disease that they would encounter later on. They sought medical consultation when the symptoms had not resolved. Like trudging in a tiptoe, the symptoms came slowly and almost silently. Since it mimicked a majority of musculoskeletal complaints that were predictably benign to a layman, the idea of taking it seriously was oftentimes laid off.

An entreaty for independence (a clash between will and physical disability): Activities like bathing, feeding, dressing up, and ambulating were vital in a patient’s daily life. The patients equated their ability to carry on these tasks with their degree of independence. All patients felt that they had become a burden to their family not only financially but also physically. Their condition demanded family members’ time and effort to assist them in doing supposedly self-care procedures. The struggle not to be a burden to their family placed a mental load on the patients themselves as they will do things on their own but their body did not permit them to do so.

Taking a toll on health (XDP and its health consequences): XDP did not only pose as a threat to the patients’ mobility but also to health consequences such as loss of appetite, weight loss, and changes in the sleeping pattern of the patients. So far, no patient reported changes in bowel and bladder function.

Psychological Manifestations

Psychological manifestations involved the following aspects:

Awareness and generic misconceptions: The transition from the generic misconceptions about the disease, which included having a mild stroke and temporary muscle spasms, towards the acquisition of knowledge that what they had was dystonia was considered crucial in the process of acceptance. Entertaining the misconceptions and ignoring the medical diagnosis was part of the denial phase.

The diagnosis and its emotional corollary: Disbelief and unacceptability were reported to be the most common initial reaction to the diagnosis. This defense mechanism was believed to take place in an attempt to ward off anxiety and other threatening emotions. A plethora of emotions followed thereafter: from anger to depression, to suicidal ideations and even acts of compensation. Feelings of frustration surfaced because of physical limitations. These findings were consistent with the results of a previous study, which showed that feelings of frustration are common among patients with chronic diseases. Unlike grief associated with non-bodily losses, chronic disabilities serve as a constant reminder of the permanency of the condition [13].

An intra-assessment (self-perception and self-esteem): Physical disability affected how the patients viewed themselves. The first reason for this deterioration was a change in their appearance and movement. The second reason was the loss of function, which led to role changes in the family and society. Their inability to perform activities of daily living and the constant need for assistance affected their self-perception. As former breadwinners, XDP also took a toll on their ego as men in the family lost their jobs and depend on others for sustenance. 

Spirituality: An increase in faith and turning to religion in times of problems were observed among patients.

Social Changes and Manifestations

Social changes involved events encompassing family dynamics and societal behavior. Three patients verbalized that their family relationship was solidified and their bond became stronger. Half of the group reported having no changes in family relations after their diagnosis while two patients claimed they were neglected by their wives and children. Family problems were present and most of these arose because of financial reasons and the attitude of the patient or caregiver.

In the context of community relationships, four patients had no changes in the way people treated them but felt ashamed about their condition. They claimed that their friends abandoned them after their illness struck. Patients considered community support an important factor in affecting their self-esteem. Social support is thought to enhance psychological well-being directly by fulfilling one’s need for a sense of coherence and belonging, thus counteracting feelings of loneliness [13].

Physical, psychological, and social aspects of coping

Physical Coping

Seeking medical consultation from either a generalist or a neurologist was a priority option for the majority. They received various medications for the control of symptoms. These included maintenance muscle relaxants. Botox injection was deemed effective for a period of two to three months. Faith healers and spiritual interventions were sought in the early course of the disease.

Psychological Coping

Several themes were identified regarding emotional coping mechanisms.

The need to share the burden and the need for reciprocation: Patients experienced the felt need to vent out emotions through words to their spouses and family members. They had expectations that their audience would listen and show genuine acceptance and concern towards these verbal expressions. Positive reciprocation through encouraging words, a caring gesture, or a simple silence brought motivation to the patients. The creation of emotional connections through spoken words was considered a means of coping.

The healing in tears and prayers: Crying was observed as an initial reaction to the diagnosis, during a depressive episode, when feeling frustrated and angry, and when thinking about the life they had before and the inevitable future they are facing. Crying as a coping mechanism was described as a way of unloading the burden and letting go of emotions, providing momentary and conditional relief. Prayer, on the other hand, was described as a source of strength, comfort, and reprieve.

Finding effective diversion tactics: Recreations including watching television and listening to the radio, playing on tablets, and betting on the lotto were considered harmless diversion tactics. Vices such as alcohol and illicit drugs were utilized by one patient as a form of coping. Diversion tactics were considered disengagement coping strategies, which sought to deal with stressful events through passive, avoidance-oriented activities.

Suicide (the contemplation, the plan, and the act): Suicide was contemplated in three situations based on the cases: (1) when patients were in a depressed mood and when they were comparing their present status with their past; (2) when they thought of being a burden to their family; and (3) when physical pain bothered them. Suicide was thought of as a means of escaping their problems.

Acceptance and coping: Acceptance was considered by patients as the only option after being diagnosed, though deemed a tedious and emotionally challenging process. Time was considered the best vehicle on the road to acceptance. It had been noted that there was no wrong or right time limit and each patient underwent a unique experience while going through the specifics of acceptance.

Social Coping

Social coping involved the following themes:

Role acclimatization was deemed necessary for successful acceptance: Role changeovers were noted as patients gradually lose their ability to function well enough to keep their jobs and be the breadwinners of their families. During the early months of diagnosis, patients got increasingly frustrated by the sudden role shifts. All patients viewed themselves as a burden to their family at some level be it a financial, emotional, or physical inconvenience. Patients strived for normalcy and desired to be treated generally like other people without the disease. Having a sense of independence negates feelings of uselessness and weakness.

The wife (a maker or breaker): The wives of the patients played a significant role in the disease experience. This included the role of a primary caregiver, a source of strength and comfort, an emotional confidante, and the family breadwinner. Attitudes toward the illness were not only important for the patient but were an integral aspect in the coping of the patient’s caregivers as well.

A picture of a father: All of the patients had children and their respective families. Being a family, these men did not only play the role of a breadwinner, but also a father, a decision-maker in the family, and a guide for their children. Only one patient affirmed that he still plays a big role in decision-making for his family. One patient claimed that children still turn to him for advice. Two patients claimed that they can effectively play the role of a father to their children, as well as a decision-maker for the family. The rest of the patients, especially those in a dependent state, passively played the role of a decision-maker but claimed that the role of a father can never be taken away from them. The physical limitations and inability to perform the same activities before the onset of illness could trigger feelings of weakness and uselessness. It was more beneficial for the patient when the family expressed love and acceptance while encouraging independence of the patient to facilitate a greater feeling of independence and confidence resulting in improved esteem.

Keeping the social circle: The patients also displayed various ways of adapting to society. Their coping responses were attributed to the way society treated them. Support and acceptance from the society aided patients in forming healthy self-perceptions and maintaining amiable social interactions with community members, while neglect and supposition of being ostracized resulted in feelings of insecurity and inferiority, and eventually to isolation.

Needs, goals, and aspirations

Needs

Financial support and a steady stream of medication supplies were priority needs. Money was described as a need than a want and was not only for their medications. The patients claimed they needed the money to compensate for the jobs they had given up. The need for medications came in tandem with the need for financial assistance.

Goals and Aspirations

Most of the patients were aware that the medication therapy they were under was simply for the purpose of controlling the spasms, relaxing the muscles, or putting them to sleep. Still, one of the patients, Paul, the young bouncer, firmly believed that he will be cured at a certain point in the future. Four patients, having the knowledge that the disease is incurable, somehow verbalized, “When I recover...” The statement was not meant to describe the fact that the patients had not understood the disease process or they were living in a sphere of denial all through this time. Instead, this implied that they hold deep within themselves a small gleam of hope: when the impossible becomes possible. Most likely these patients experienced “magic hope,” which referred to wishful expectations that would be fulfilled by external forces such as fate. Six patients, when asked what their dreams were, replied “I don’t have one for myself, but as for my family...” Three recounted their dreams before the disease and their dreams when they can recover. One patient felt that his dreams had all been fulfilled and he was quite content with his life.

Fears

Some patients confided their worst fear that the disease would progress, leading them to a lifetime of dependency and complete disability. The thing with this particular fear was the unpredictability of its commencements. One would never know which region of the body the dystonia would strike next, how many months or years would pass when the symptoms would start to manifest, and how severe and disabling these symptoms would become. These thoughts of fear of oblivion played around their minds.

Thoughts of the Future

Being grateful and contented about the life they lived, despite having the disease, was a rare instance among the patients. But two patients said that they had lived their lives to the fullest and were happy for the extra years that they had. They being grateful shed new light on the notion that having dystonia was a curse and a burden. They believed that everything was about perspective. When patients were asked about their dreams and views of the future, the majority decided they wanted something positive to happen, if not for them, for their families. Most of them desired a bright future for their children and endeavored to be strong despite their disease for their kids’ sake. One patient wanted to die because he felt that he had been a burden to his family.

Quality of life: its eventful decline

**Figure 2:**
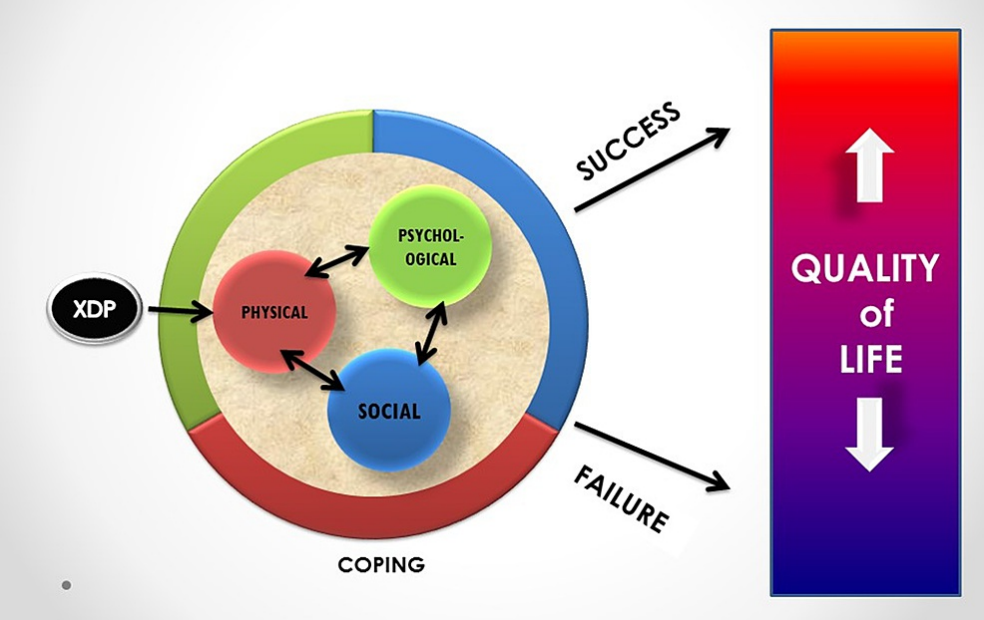
Diagram of findings From the leftmost corner, the diagram begins with a circle representing the disease XDP. An arrow from the disease directly points to a small red circle representing the physical attributes of the disease. These attributes include torticollis, blepharospasm, limb torsions, and other manifestations. A double-headed arrow shows that the physical attributes interact with two other circles in a reciprocal manner. The disease itself has no direct impact on the blue and green circles representing social and psychological manifestations, respectively. Rather, these aspects are brought into play because of the presence of the physical manifestations of the disease. A bigger circle surrounding the three small circles represents coping. The coping circle is color-coded similarly with the smaller circles, representing, physical, social, and psychological coping. The coping circle buffers the effects of the disease on a patient’s perception of quality of life. Success and failure in coping predict at what end of the spectrum the patient’s quality of life would fall into. XDP, X-linked dystonia-parkinsonism.

The QoL is a constantly shifting measure, from positive to negative, from excellent to poor. It can be seen as a spectrum. A spectrum will depict that a patient’s QoL is not a constant point that one can accurately plot (Figure 2).

Most patients reported a decline in their QoL, which may be attributed to their inability to perform work, the loss of independence, and the pain and physical exhaustion felt due to their situation. Furthermore, poor symptom management and medical treatment, financial instability, and/or lack of concern of the family were also attributable factors in the decline. This deterioration in QoL was not only because of physical aspects but also because of social and self-esteem changes brought about by the onset of their illness (Table 3). Only two patients perceived a very good QoL and both had the disease for more than 12 years, accepted their limitations, and found means to enjoy their simple pleasures.

Implications: theoretical framework

A patient’s QoL, according to the results of the study, was determined by multifactorial situations involving his physical disabilities, emotional and mental status, social relationships and family support, financial and medical standing, as well their self-perception and personal integrity.

Engel’s biopsychosocial model describes human functioning in the context of disease and illness as an interplay of three systems, namely, biological, psychosocial, and social systems. In this study, the patient’s QoL was determined by multifactorial situations involving his physical disabilities, emotional and mental status, social relationships and family support, financial and medical standing, as well his self-perception and personal integrity. From a practical standpoint, it gives us an opportunity to understand each and every patient’s subjective experience as an essential contributor to health outcomes and, ultimately, his QoL [14].

For clinicians and healthcare members alike, an important implication of the theory in relation to the results is the growing need for a holistic approach to patient care. Patient-centered care beyond medical diagnosis and treatment calls for physicians to elicit psychosocial and social situations that could allow patients to elaborate their personal subjective perspectives. Most of the patients in our study had entertained suicidal thoughts more than once through the years they were living with the disease. It is of vital importance that the physician would gain a shared understanding with the patient regarding his or her disease experience and be able to deal with it. The results and the theory calls for physicians and researchers who are mindful, tactful, observant, and sensitive of the patient.

Abraham Maslow’s hierarchy of needs focuses on humanism. Maslow stated that certain circumstances can make a person’s level of motivation regress like a traumatic life experience or compromised health [15]. Indeed, most of the patients experienced stagnancy in the ladder of motivation and the fulfillment of needs. However, some patients claimed that they have come to a point of contentment. The theory, on the other hand, suggests that when basic needs are met, higher needs emerge. The results of the study showed that patients have physical, psychological, spiritual, and social needs that they continually strive to achieve. The holistic care approach of the physicians will always be the best way of treatment. Patients deemed it necessary that their physical needs are met first, which include alleviation of pain, independence, and mobility.

The five stages of grief by Kubler-Ross shows a series of emotional stages that a person experiences when faced not only with imminent death but also with other extreme, awful fates [16]. Results of the study showed that patients did experience the five stages at certain points starting from their diagnosis. Denial, anger, bargaining, depression, and acceptance were sometimes seen as overlapping and interchanging phases. As patients go through the stages, the lack of a rigid wall between the stages allowed the patient to go back and forth. Emotional lability, with its constant need for a watchful eye and support from family members, was greatly emphasized.

Lazarus and Folkman’s transactional model’s key premise is that primary appraisal, secondary appraisal, and coping strategies mediate the relationship between the stressor and the individual’s stress outcomes [17]. During the process of primary appraisal, patients would be having thoughts of what ill health would bring to their jobs and their families, still, on the premise that the disease would be temporary. The secondary appraisal would be triggered by the clinical diagnosis of dystonia. Patients would have a lot of thoughts in mind to combat the impending threat. These preparations, be it physical, psychological and mental, spiritual, social, or financial, are evaluated to be sufficient or insufficient by the patient. Coping responses are initiated after the cognitive appraisals and the eventual psycho-physiological experiences (stress outcomes) of this potentially stressful event depend on the effectiveness of one’s cognitive appraisals and coping processes.

Coping had always been a relative process for the patients. The constant need for stability in an otherwise stressful life keeps the greatest burden on the patient’s ability to cope. The inability to cope predicted stressful outcomes and a decline in the QoL. Strengthening the coping abilities and helping patients find effective coping strategies should be kept in mind by the caregivers in dealing with these patients.

Roy’s adaptation model presents the person as a holistic adaptive system in constant interaction with the internal and external environment [18]. Adaptation leads to optimal health and well-being and to QoL. Indeed patients’ variation in responses to their diagnosis of dystonia can be attributed to their innate coping skills. Patients over time acquired coping skills that can handle the chronicity of the disease process. These include physical coping strategies like body positions that make them comfortable and what aggravates the torsions; and psychological coping strategies, which include thoughts towards their family and optimism for their children’s brighter future.

The implications of the theories, as a summary, are focused on the interaction of the different factors that affect disease perception. These include a biopsychological approach to the disease, patient’s needs, emotional manifestations, stress, coping, and adaptation.

## Conclusions

This study concludes that QoL is a fluid state that can encompass both positive and negative aspects of life at the same time. QoL, for most of the patients, is most often perceived as declining, moving downwards across the spectrum. The downward movement can be gradual for some or rather hasty for others depending on the factors that played a part in their lived experiences. This can be attributed to the unavoidable disease progression, the continuing appearance of new symptoms, and the steady loss of ability to function independently. The perception of the QoL can be modified in just a couple of months to years and this likely depends on the rate of the disease progression and the onset of new symptoms that add to the set of physical limitations to the patient. Therefore, a patient’s QoL is subjective, predictable, fluctuating, and modifiable.

XDP patients had individualized experiences with the disease. The patient’s QoL was affected by a different interlocking chain of factors. The multidimensional aspects of the QoL should be placed under constant check and balance; the family and health workers alike should be the accountants. Finding meaning to their QoL did not only rely on physical relief of symptoms, but on gaining social acceptance, independence, and life-long support and love.
